# One-step synthesis of Pt/a-CoO_x_ core/shell nanocomposites

**DOI:** 10.1186/s42649-019-0016-2

**Published:** 2019-11-14

**Authors:** Daewoon Kim, Sung Joo Kim, Jong Min Yuk

**Affiliations:** Department of Materials Science and Engineering, KAIST, Daejeon, 305-701 South Korea

**Keywords:** One-step synthesis, Nanocomposites, TEM, EDS

## Abstract

Herein, we synthesize a core/shell Pt/a-CoO_x_ nanocomposite via one-step synthesis using a strong reaction agent of borane t-butylamine(BBA) at 200 °C. Transmission electron microscopy study shows that the morphology of nanocomposites is controlled by the stirring time and perfect core/shell structure is formed with over 7 days stirring time.

## Introduction

Nanocomposites containing Pt have attracted great attentions due to their excellent catalytic, electric and magnetic properties. (Peng and Yang, 2009; Li et al. 2015; Zhang et al. 2013; Wang et al. 2015; Esfahani et al. 2010; Wang et al. 2010) Since these properties closely intertwine with their size, shape and composition, designing nanocomposites is critical to their chemical, electrical and energy applications. (Pushkarev et al. 2012; Vidal-lglesias et al. 2012; Mostafa et al. 2010; Wang et al. 2013) Among diverse nanocomposites, core/shell structures of Pt/transition metal oxide, such as Pt/Fe_2_O_3_, FePt/Fe_3_O_4_ or Pt/CoO, not only show remarkable magnetic properties, but also contain small amount of expensive Pt. (Alayoglu et al. 2008; Tao et al. 2008; Zhao and Xu, 2006; Zhou et al. 2005; Teng et al. 2003; Zeng et al. 2004; Yin et al. 2004; Habas et al. 2007) Traditionally, the core/shell structures have been synthesized by a two-step growth method. (Tao et al. 2008; Liu et al. 2005; Yu et al. 2014) Core nanoparticles are synthesized first as seeds, followed by growth of the shell around the core. However, the two-step growth technique typically suffers from low yield because the synthesized core particles are not well dispersed and shell materials independently coalesce each other instead of adhering to the core. In our study, we report a (scanning) transmission electron microscopy ((S)TEM) study of Pt/amorphous cobalt oxide (a-CoO_x_) nanocomposites growth by one-step heating synthesis.

### Experiments

**Fig. 1:**
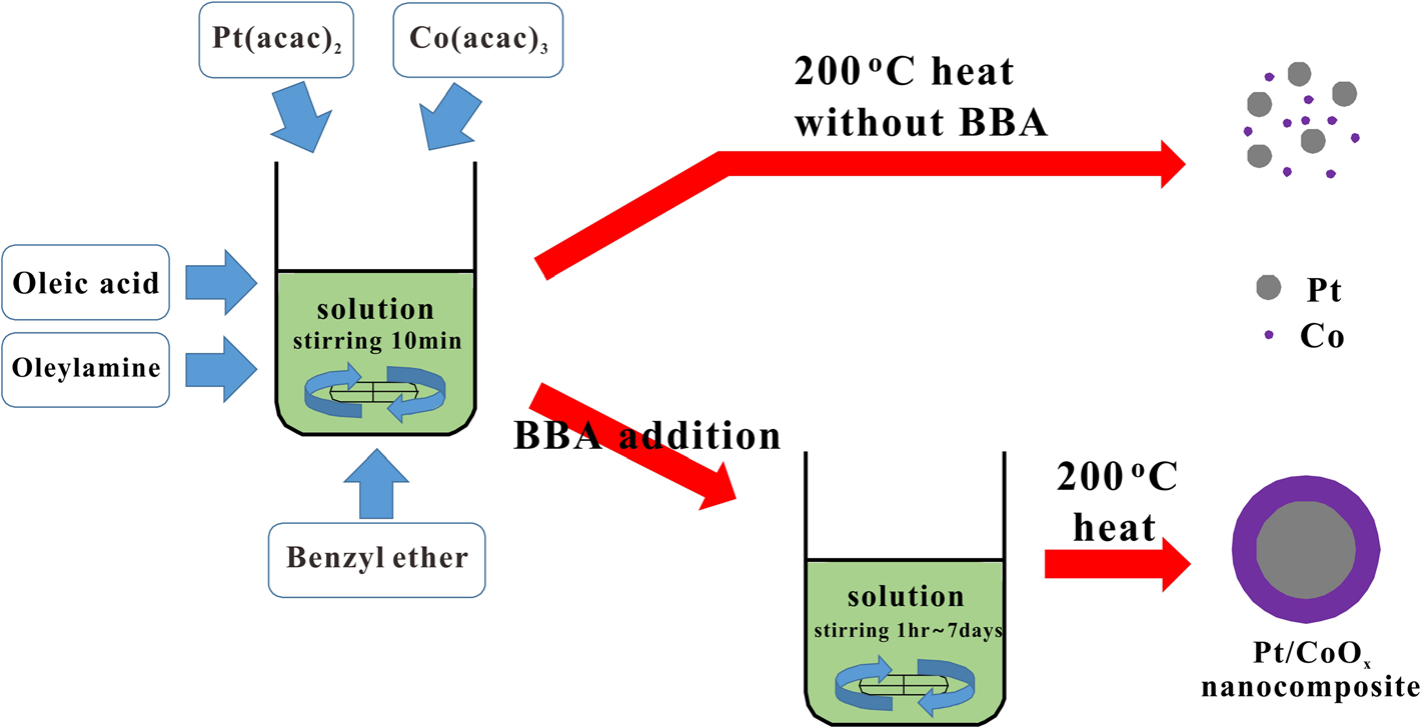
Schematic illustration of the synthesis procedure for Pt/CoO_x_ nanocomposite

The nanocomposites are synthesized with platinum(II) acetylacetonate(Pt(acac)_2_) (97%), cobalt(III) acetylacetonate(Co(acac)_3_) (98%), oleylamine(98%), oleic acid(90%), benzyl ether(98%), and borane tert-butylamine(97%) from Sigma-Aldrich Co.. 1 M Pt(acac)_2_, and 3 M Co(acac)_3_ were dissolved in 0.6 mL oleic acid, 6 mL oleylamine and 53.4 mL benzyl ether(total 60 mL solution). The solution is heated to 50 °C under magnetic stirring for 10 min. Here, we add 1 M borane t-butylamine (BBA), which is a more powerful reaction agent than oleylamine or oleic acid. (Yu et al. 2014) (Fig. 1) Then the chemicals are further heated to 200 °C and kept for 2 h using autoclave oven. After the solution is cooled to room temperature, the nanocomposites are obtained after several washes with 40 mL ethanol by centrifuging at 3000 rpm for 10 min and dried under vacuum. The final products are dispersed in toluene. According to the stirring time of the solution, we analyze the morphology of the synthesized nanocomposites using a TEM. The TEM imaging is performed using JEOL ARM200F operated at 200 kV in conjunction with a Bruker Quantax energy-dispersive X-ray spectroscopy (EDS) detector.

## Results and discussion

**Fig. 2:**
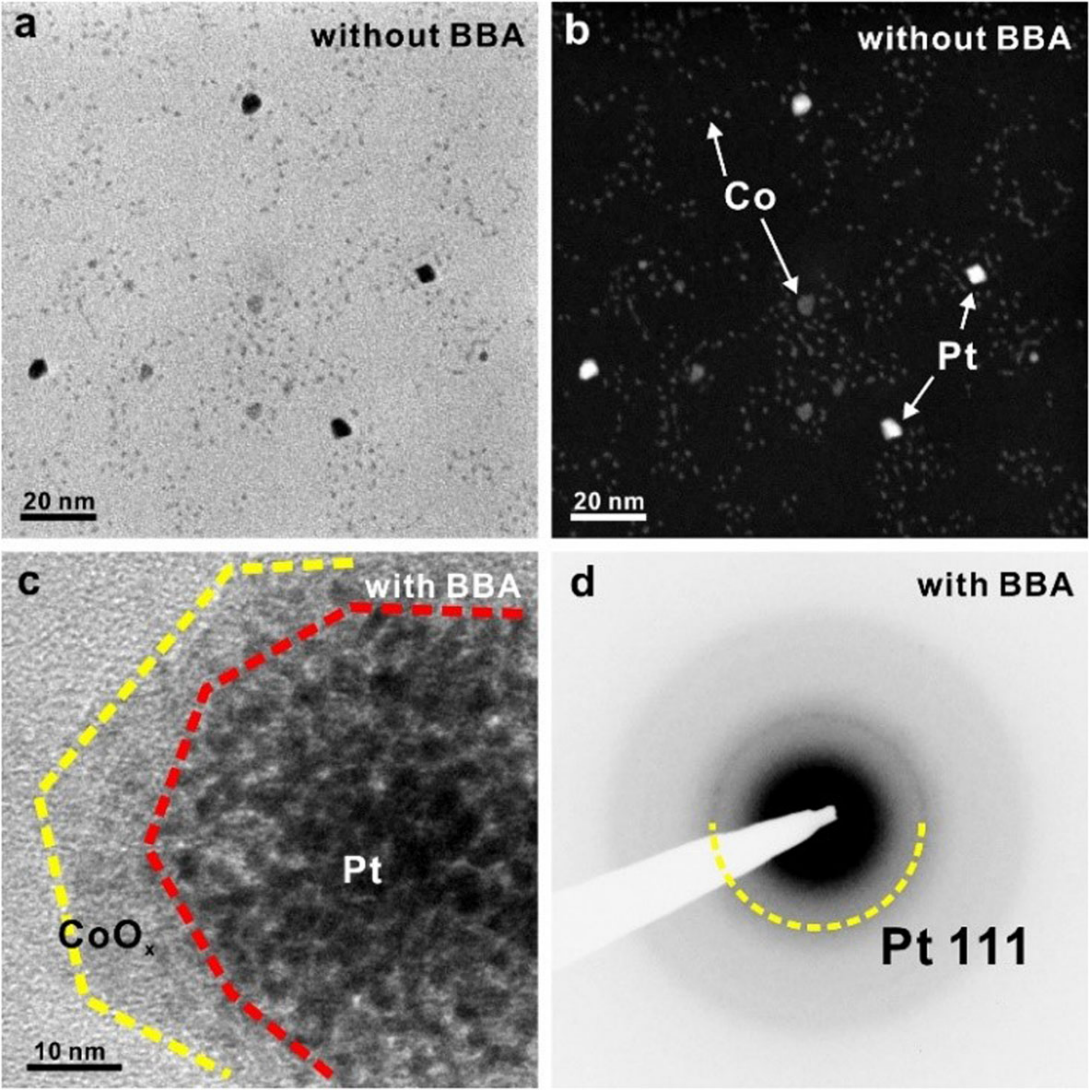
TEM image of Pt/CoOx nanocomposite (**a**) Bright-field and (**b**) dark-field STEM images of Pt and Co nanoparticles without using a BBA additive during synthesis. **c** TEM image of synthesized Pt/a-CoO_x_ core/shell nanocomposites when using a BBA additive. **d** Selected area electron diffraction (SAED) pattern of area (**c**)

TEM/STEM images in Fig. 2 show the effect of a BBA additive on a synthesis of Pt/CoO_x_ nanocomposites. Without BBA addition into the precursor solution, the synthesized Pt and Co are formed separately with forming a compound. (Fig. 2)a Using the Z(atomic number)-contrast dark-field STEM imaging, 5 nm sized bright nanoparticles are likely Pt while the rests with a size distribution of 0.96 ± 0.56 nm are Co. (Fig. 2b, Additional file 1: Figure S1) In the synthesis, the color of the reaction solution changes from yellow to black at around 140 °C when Pt(acac)_2_ only added in the solution. On the other hand, the color of the solution does not change at around 200 °C for 2 h when Co(acac)_3_ only added. However, in our study, the reduction reaction of Co(acac)_3_ is observed at 200 °C when two precursors are simultaneously added. This shows that pre-synthesized Pt nanoparticles act as catalysts to lower the reduction temperature of Co(acac)_3_ to below 200 °C. However, the number of formed Pt nanoparticles is not sufficient enough for Co reduction to grow Co nanoparticles, making Pt and Co form separately. Thus, we recognize that Pt nanoparticles need to be reduced at much lower than 140 °C in order to produce a composite of Pt and Co. Figure 2c shows the bright field TEM image of Pt/CoO_x_ nanocomposites with the addition of BBA. By adding BBA inside the solution, the color of the solution changes to black at 60 °C. This shows that reduction temperature of Pt(acac)_2_ is lowered below 60 °C. This change makes Pt nanoparticles form inside the solution much more than the one without BBA to ultimately have the reduced cobalt clusters increased. With BBA, the synthesized nanostructure forms a core-shell structure. Pt nanoparticles are well distributed with amorphous shells wrapped around them. In a selected area electron diffraction (SAED) pattern of the nanocomposite, polycrystalline Pt is formed in a core with an amorphous shell formed outside. (Fig. 2d).

**Fig. 3:**
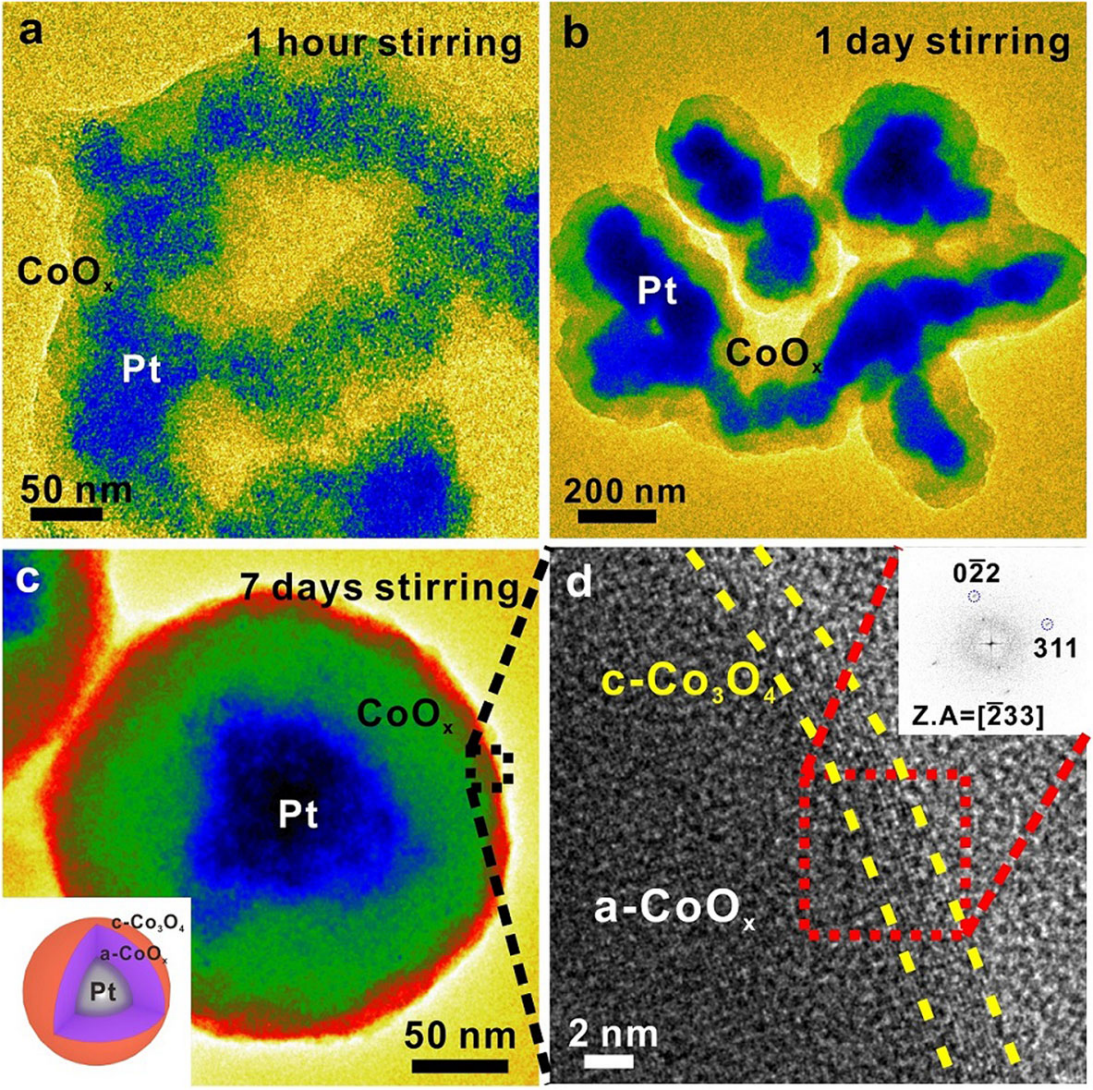
Bright-field TEM images and high-resolution TEM analysis of the one-step synthesized Pt/CoO_x_ nanocomposites under various stirring conditions (**a**) 1 h, (**b**) 1 day and (**c**) 7 days. Inset: Schematic illustration of the synthesized Pt/CoO_x_ core/shell nanocomposite. **d** Zoomed in high-resolution image of a square box in (c). Inset: fast Fourier transform (FFT) pattern of a red square in (**d**)

In order to change the morphology of nanocomposites, we further modify the synthesis of Pt/CoO_x_ nanocomposites by adjusting the stirring time at 50 °C (Fig. 3). Under 1 h stirring, Pt nanoparticles are found to spread widely while the shell structure is grown to surround them (Fig. 3a). Under 1 day stirring, Pt nanoparticles coalesce to form a larger nanocomposite core than that with 1 h stirring (Fig. 3b). Finally, under 7 days stirring, Pt nanoparticles are aggregated to a size in between 50 and 100 nm, while an amorphous shell surrounds uniformly to form a perfect spherical shape (Fig. 3c). The overall size of core/shell nanocomposites is 100~200 nm.

Although BBA originally reduces Pt precursors at 60 °C, stirring at 50 °C for a sufficiently long time after adding BBA produces agglomerated Pt seeds without growth due to low temperature. When the temperature is raised, the seeds grow and accumulate in the core. Further increasing the temperature reduces the Co precursor to form an oxide shell around the core. Figure 3d shows the high-resolution TEM image of the outer surface of the synthesized core/shell nanocomposites. It is clear that the shell is composed of an amorphous structure but with 3 nm crystalline CoO_x_ formed on the outermost surface. A corresponding fast Fourier transform (FFT) image identifies the structure to be Co_3_O_4_. This suggests that the surface of the nanocomposites transforms from amorphous to crystalline by exposing it to air.

**Fig. 4:**
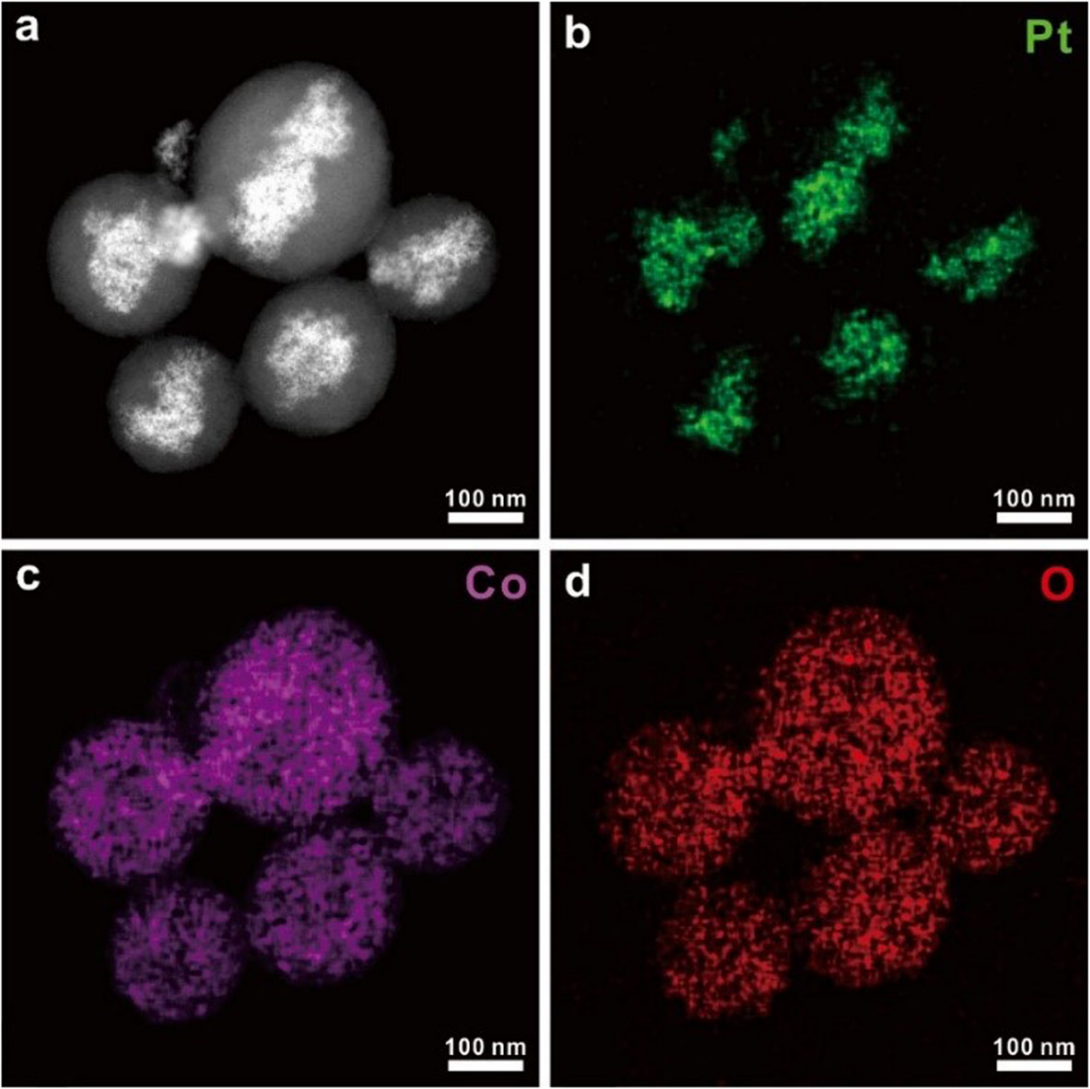
EDS mapping of Pt/CoOx nanocomposites (**a**) STEM dark-field image and (**b**-**d**) the corresponding EDS maps of Pt/CoO_x_ nanocomposites after 7 days of stirring

Figure 4 shows the STEM EDS mapping for composition analysis of core/shell nanocomposites. At the nanocomposite core, it is confirmed that the bright area in a STEM image in Fig. 4a consists of Pt while (Fig. 4b) Co and O are shown at the shell (Fig. 4c, d). The quantitative analysis clearly suggests that the shell structure is Co_3_O_4-x_ (Additional file 1: Figure S2). In Fig. 4b, the morphology of Pt is distributed like a band in a specific direction. However, in Fig. 4c and Fig. 4d, Co and O have a spherical shape. This result shows that the magnetic stirring caused the aggregation of Pt seeds to be banded in a specific direction, and then the CoO_x_ shell was formed after temperature rises.

## Conclusion

We synthesize the Pt/a-CoO_x_ core/shell nanocomposites using one-step method. BBA is added to allow reduction of Pt and Co precursors to occur at low temperature. Low temperature stirring is performed to change the morphology of nanocomposites. After 7 days stirring, the core/shell nanocomposites are synthesized in which Pt nanoparticles are formed in the core with amorphous/ crystalline cobalt oxide formed at the shells.

## Supplementary information

**Additional file 1: Figure S1.** Size distribution of cobalt nanoparticles in Fig. 2b. average size of cobalt nanoparticles is 0.96 nm, and standard deviation of the sizes is 0.56 nm. **Figure S2.** Quantitative EDS graph of the entire particle in Fig. 4. Atomic ratio of cobalt and oxygen is 45:55, seems very close to Co_3_O_4_.

## Data Availability

The datasets used and/or analyzed during the current study are available from the corresponding author on reasonable request.

## Abbreviations

(S)TEM: (scanning) transmission electron microscopy
acac: acetylacetonate
BBA: Borane t-butylamine
BF: Bright-field
DF: Dark-field
EDS: Energy-dispersive X-ray spectroscopy
FFT: Fast fourier transform
SAED: Selected area diffraction pattern
