# Supplementary material for: One-step synthesis of Pt/a-CoOx core/shell nanocomposites
Source: Appl Microsc. 2019 Nov 14;49:12. doi: 10.1186/s42649-019-0016-2 (PMC7818283; doi:10.1186/s42649-019-0016-2)
Supplement: Supplementary file 1 — Additional file 1: Figure S1. Size distribution of cobalt nanoparticles in Fig. 2b. average size of cobalt nanoparticles is 0.96 nm, and standard deviation of the sizes is 0.56 nm. Figure S2. Quantitative EDS graph of the entire particle in Fig. 4. Atomic ratio of cobalt and oxygen is 45:55, seems very close to Co3O4. [file 42649_2019_16_MOESM1_ESM.docx]

**One-step synthesis of Pt/a-CoO_x_ core/shell nanocomposites**

Daewoon Kim^1^, Sung Joo Kim^1^, and Jong Min Yuk^1^*

^1^ Department of Materials Science and Engineering, KAIST, Daejeon 305-701, Korea


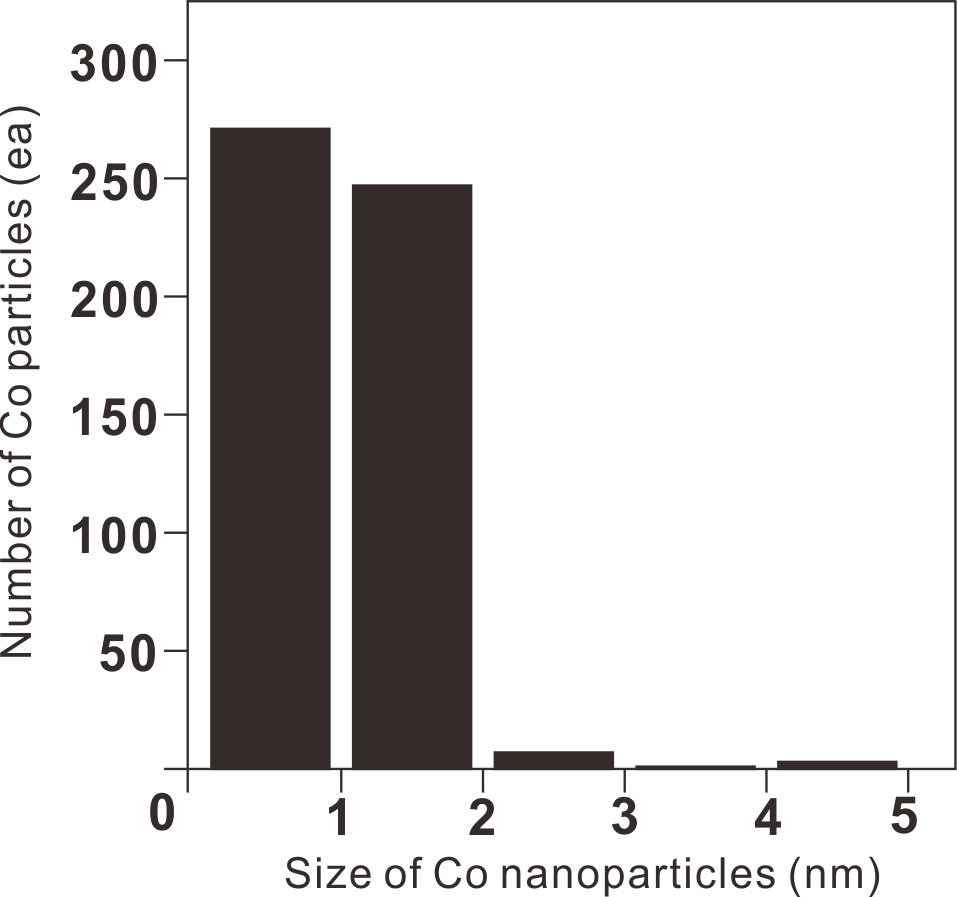


**Figure S1.** Size distribution of cobalt nanoparticles in figure 2b. average size of cobalt nanoparticles is 0.96 nm, and standard deviation of the sizes is 0.56 nm.


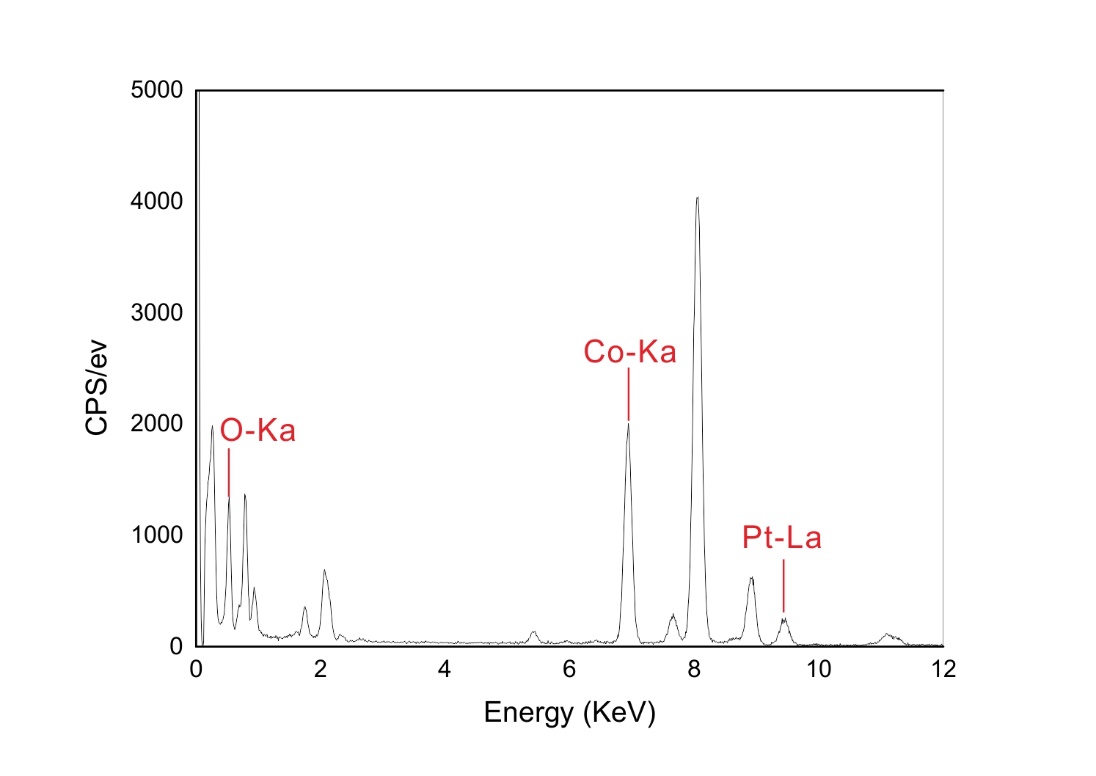


**Figure S2.** Quantitative EDS graph of the entire particle in Figure 4. Atomic ratio of cobalt and oxygen is 45:55, seems very close to Co_3_O_4_.
